# Intelligent Matching Algorithm with Density-Based Clustering for UAV Swarm Networking

**DOI:** 10.3390/s26175588

**Published:** 2026-09-03

**Authors:** Leyi Kong, Dong Guo, Jiaqi Xu, Fu Wang, Jiahui Wu, Xiangjun Xin

**Affiliations:** 1 School of Information and Electronics, Beijing Institute of Technology, Beijing 100081, China; akongleyi@163.com (L.K.); xinxiangjun@bit.edu.cn (X.X.); 2 School of Science, Beijing University of Posts and Telecommunications, Beijing 100876, China; xjq1997@bupt.edu.cn (J.X.); amy_wjj025@126.com (J.W.); 3 School of Electronic Engineering, Beijing University of Posts and Telecommunications, Beijing 100876, China; wangfu6672@gmail.com

**Keywords:** density-based clustering, intelligent matching game, unmanned aerial vehicle

## Abstract

As an emerging and powerful technology, unmanned aerial vehicles (UAVs) have tremendous potential applications in real-time monitoring, instant communication, data transmission, and more, providing ground terminal users with more efficient services and support. In this paper, we analyze the optimal networking scheme for user association with UAVs based on matching algorithms, which offer higher throughput and satisfaction. Particularly, to minimize algorithm complexity and enhance the efficiency of user devices, we propose a novel approach for stable UAV–user device pairing. This framework combines the concepts of density-based clustering algorithms and utility-driven matching algorithms. Firstly, we address the issue of large-scale scenarios with numerous and unevenly distributed user devices by proposing a clustering algorithm. This clustering algorithm divides the geographical area into multiple grids and clusters based on local density and relative distance within each grid. Next, we introduce a hierarchical matching game, where user clusters and UAVs are the players in the game. Each player ranks the other based on their individual utility functions, constructing preference lists of UAVs for users and vice versa. The network resource balancing and efficiency maximization are achieved through the matching process of bilateral selection. Simulation results demonstrate that this method exhibits low average required transmit power per user and the highest throughput among the five compared schemes under hotspot user distributions.

## 1. Introduction

In recent years, natural disasters have been occurring frequently, posing severe threats to people’s lives and properties and often resulting in significant damage to communication infrastructure [1]. Equipment such as base stations and communication towers may be destroyed or rendered inoperable, leading to a sharp decline in communication quality, significant increase in communication latency, or even communication breakdown, thereby causing significant difficulties for rescue and recovery efforts. In the process of addressing these challenges, traditional fixed communication networks often appear inadequate. Unmanned aerial vehicles (UAVs), with their advantages of miniaturization, low cost, and high dynamics, have attracted widespread attention from various sectors of society, providing a new generation of solutions [2]. UAVs are lightweight, weighing only a few kilograms, enabling them to achieve high-speed movement with lower energy consumption, demonstrating excellent flexibility and maneuverability. Additionally, UAVs are typically equipped with multiple cameras and sensors, enabling effective collection of ground and aerial data, facilitating efficient data acquisition and transmission [3].

With the continuous integration of technologies such as artificial intelligence, the Internet of Things (IoT), and cloud computing, UAVs are no longer standalone units but have become part of a vast network of unmanned swarms [4]. As the scale and complexity of these networks increase, new challenges arise regarding network transmission latency, resource allocation, and more. Effectively organizing and managing these networks, which comprise diverse technologies including UAVs and IoT devices, to ensure their efficient and stable operation has become an important and complex task [5]. UAV swarms’ intelligent networking technology offers effective solutions to address these challenges. Through intelligent networking, unmanned swarms can automatically configure network parameters, dynamically adjust resource allocation, and optimize network paths in real time, thereby effectively reducing transmission latency and improving resource utilization efficiency. Additionally, intelligent networking technology enhances the network’s self-healing capabilities and robustness, ensuring stable operation even in the face of node failures or external interference [6]. Therefore, unmanned cluster intelligent networking technology is not only crucial for enhancing the efficiency and stability of cluster network operations but also serves as a significant driver for the deep integration of technologies such as UAVs, IoT, and cloud computing, enabling broader application scenarios. However, existing clustering-based association methods often face high computational complexity or rely on simplified assumptions, which limits their use in dense and unevenly distributed user environments. We therefore design a density-aware clustering and matching framework for practical UAV swarm networks.

**Figure 1:**
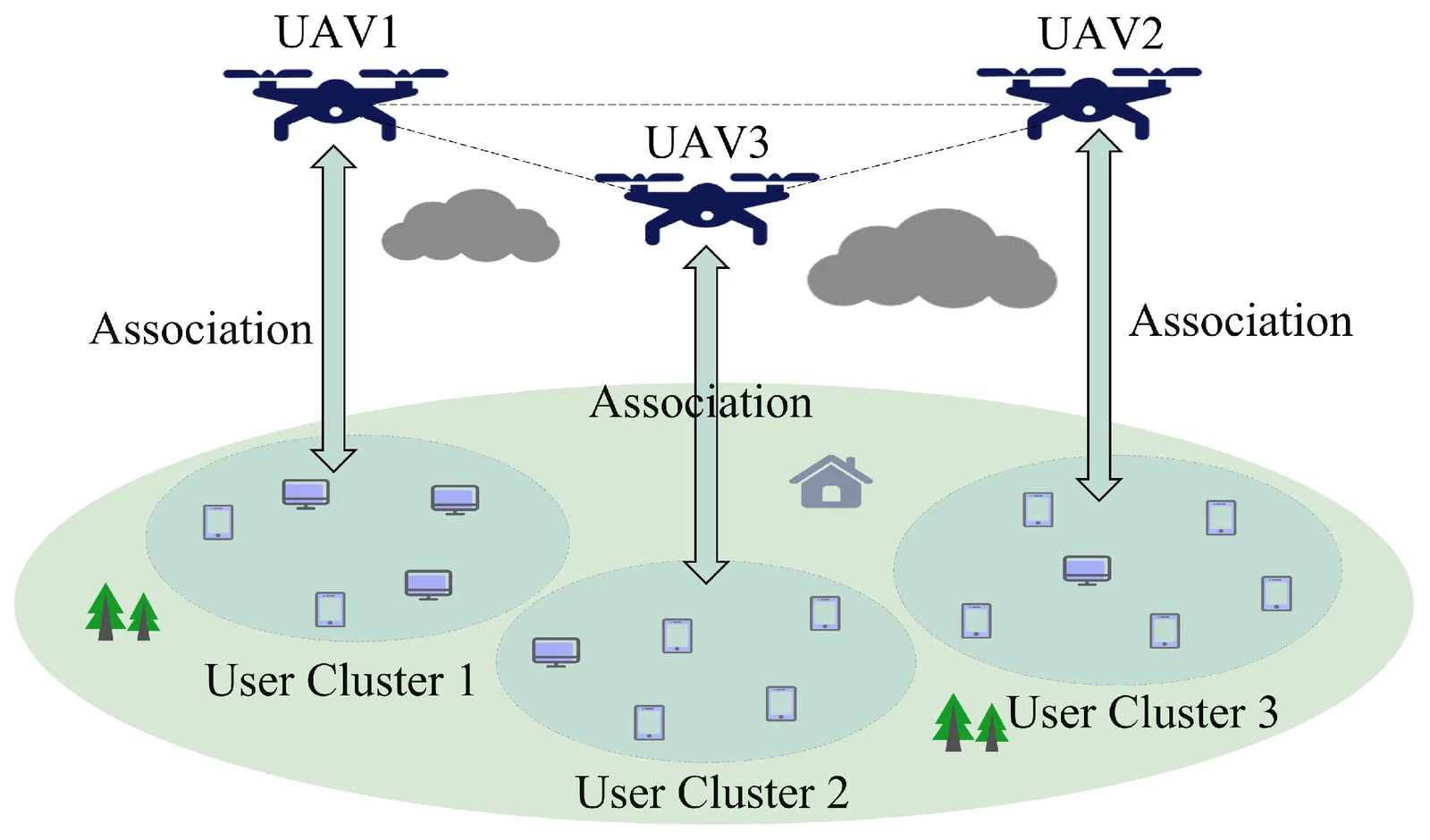
Intelligent matching architecture with density-based clustering for UAV–user association.

In this work, our goal is to analyze and design a set of associations between UAVs and user devices to provide reliable connections with low complexity and high communication performance. In practical networks, user distribution is often uneven, necessitating that the network’s organizational structure can flexibly adapt to user distribution to achieve effective resource utilization and optimize network performance. Therefore, this paper designs an efficient and highly accurate clustering algorithm for unmanned swarms’ users, identifying dense user distribution areas based on their geographical locations. Subsequently, an intelligent and efficient cluster matching method is designed to maximize resource utilization, enhance network service quality, and improve work efficiency. As shown in Figure 1, users in the network are randomly distributed within a predefined geographical area, with each UAV hovering at a fixed height to provide support services to users.

This paper proposes an intelligent networking method for UAVs and terminal devices based on density clustering and intelligent matching, aiming to enhance network service quality and efficiency by optimizing the network structure given the positions of UAVs and user devices. Our main contributions can be summarized as follows:

- We introduce a density-based clustering algorithm for unmanned swarms’ users to address the issue of uneven user distribution. This algorithm partitions the geographical area into grids and selects several grid units with the highest decision values as cluster centers. Subsequently, clustering is performed based on the distribution of user positions and their relative distances to the cluster centers, followed by verification of the clustering results.
- We propose an intelligent matching method for unmanned swarms based on deferred acceptance, achieving stable user association with UAVs based on the clustering results mentioned above. This method establishes a hierarchical resource allocation framework where UAVs and user clusters construct preference lists and achieve stable matching according to their own service demands and capabilities.
- The experimental results show that our method has lower runtime than DPC at large *N* and lower average required transmit power per user than the random baseline. This comes from grid-based clustering, which replaces the $O(N^{2})$ pairwise-distance computation with $O(N\cdot K+G^{2})$ operations over *K* clusters and *G* grid cells.

The rest of this paper is organized as follows. In Section 2, we elaborate on the related work. In Section 3, we describe the system model and propose optimization objectives for both UAVs and users. In Section 4, we introduce a density-based clustering algorithm for users, obtaining user clusters equal in number to the UAVs. Section 5 provides the algorithm for matching users with UAVs. In Section 6, we present the simulation results, and the conclusion is in Section 7.

## 2. Related Work

In this section, we elaborate the related work of UAV network, including user association in UAV network and networking technologies.

### 2.1. User Association in UAV Network

The user association in UAV network has attracted widespread attention due to its direct impact on spectral efficiency, energy consumption, and overall quality of service (QoS) in dynamic, heterogeneous environments. The inherently dynamic nature of UAVs and the resulting overlapping coverage areas pose critical challenges to optimal user allocation. The following works represent recent advancements in these areas, offering diverse solutions to optimize user association and enhance overall network performance in UAV network.

In [7], the authors conducted a comprehensive analysis of the multi-domain autonomous unmanned command information system from a security perspective, playing a crucial role in unmanned command flow and acceleration. In [8], a top-down hierarchical approach was introduced by the authors. That method decomposed the overall mission into phases, tactics, and algorithms to enhance the effectiveness of the general cluster mission pattern framework and promote the reusability of architecture in group tasks. In [9], the authors studied a decentralized group navigation method based on real-time detection using onboard RGB cameras to achieve scalable and highly reliable, unmanned swarms’ system navigation on candidate target directions. The authors in [10] proposed a trust model based on a multi-criteria fuzzy approach to enhance network adaptability. In [11], the authors introduced a novel cluster network architecture with software-defined networking (SDN) cluster controllers and collaborative controllers for hierarchical management and unified scheduling. Different weights were assigned to various traffic based on their sensitivity to latency and importance levels. In [12], the authors presented a digital-twin-based unmanned swarms’ intelligent collaboration framework to accurately reflect the unmanned swarm and monitor its entire life cycle with high fidelity. Simultaneously, a decision model integrating machine learning algorithms was built to explore global optimal solutions. In [13], the authors proposed a multi-state network reliability assessment model based on information exchange capacity, along with an improved fast state-space decomposition boundary algorithm. That model enhanced the reliability assessment of unmanned swarms’ information exchange networks. In [14], the authors introduced a distributed network architecture combining autonomous navigation systems and collaborative control systems to meet the development of functional modules under software-defined patterns.

### 2.2. Networking Technologies in UAV Network

To enhance the performance and reliability of UAV swarms’ networks, advanced networking technologies play a crucial role. These technologies not only enable efficient user association and resource allocation, but also support dynamic clustering, energy management, and robust connectivity in rapidly changing environments.

In [15], the authors studied user devices within UAV coverage areas during disaster recovery, using clustering methods to improve wireless coverage service detection capabilities. In [16], the authors researched UAVs and edge devices, proposing a novel algorithm combining wireless link disruption probability and K-means clustering to compute the optimal number of UAVs needed to cover specific areas. In [17], the authors investigated UAV-assisted mobile communication, presenting a modular-based improved Louvain method for dynamic clustering to reduce transmit power of mobile devices and UAV energy consumption. In [18], the authors examined energy-saving group intelligent clustering algorithms in UAV networks for emergency communication, selecting cluster heads based on enhanced particle optimization. In [19], the authors studied interference-aware UAV routing and IoT device clustering algorithms, while [20] addressed the coverage problem with UAVs as mobile receiver nodes, proposing a dynamic distributed clustering approach. In [21], the authors proposed a mobility and location-aware stable clustering mechanism, selecting stable cluster heads based on nodes’ maximum coverage probability and k-means algorithm, introducing an active backup scheme to locate when the current cluster head exits the network, reducing network overhead. In [22], the authors introduced a Bayesian classifier mechanism to select the optimal node as cluster head, reducing system deployment time and enhancing system efficiency and usability. In [23], the authors investigated networking schemes for task-oriented cluster networks to reduce energy consumption of battery-powered UAVs and prolong the network’s lifespan. In [24], the authors proposed an energy and mobility-aware secure clustering algorithm to address UAV’s rapid mobility issue. In [25], the authors combined moth flame optimization algorithm and a variant of K-means method for network construction and node deployment, reducing cluster construction time and extending cluster lifespan. In [26], the authors presented a solution combining particle swarm optimization algorithm and K-means algorithm to jointly optimize the position of small UAV cellular base stations and find their minimum quantity. In [27], the authors researched dictionary trie structure prediction algorithms and link expiration time mobility models to address the issue of UAV high mobility. In [28], the authors introduced a UAV swarm network clustering algorithm based on deep reinforcement learning to improve average link retention time. In [29], the authors investigated cluster networking methods based on distance and energy constraints for source signal forwarding. In [30], the authors studied UAV network deployment with dynamic weighted clustering algorithms, analyzing network survival performance when UAV node energy was depleted or accidents occurred.

The above works have advanced UAV networking and clustering techniques, but most existing clustering-based approaches either require $O(N^{2})$ pairwise distance computations or lack a matching mechanism for UAV–user association [31,32,33]. We combine grid-based density clustering with a utility-driven matching game to address both issues.

## 3. System Model

In this section, we propose the network model and optimization function in our problem.

We consider a network model consisting of heterogeneous UAVs and users, where a set $I=\{I_{1},I_{2},\ldots ,I_{N}\}$ of *N* users are randomly distributed within a geographical area with location $\left(x_{i},y_{i}\right)$. Set $U=\{U_{1},U_{2},\ldots ,U_{M}\}$ comprises *M* heterogeneous UAVs that hover at fixed heights *h*, aiming to provide support services to users. In this system, users need to be divided into *M* clusters based on their geographical locations to accomplish the final UAV–user association.

### 3.1. Air-to-Ground Channel Model

When low-altitude UAVs transmit data, obstacles on the ground such as buildings and trees may obstruct direct line-of-sight paths, affecting signal propagation. Therefore, models for both line-of-sight (LoS) and non-line-of-sight (NLoS) propagation paths must be considered to analyze signal characteristics. LoS communication provides a clearer and more stable signal path, while NLoS communication introduces signal reflections, diffractions, and scattering due to the presence of obstacles, increasing the complexity and uncertainty of signal paths [34]. The expression for the probability of line-of-sight links in the air-to-ground channel between UAV $U_{m}$ and user $I_{n}$ is:

(1) $$p_{m,n}^{\mathrm{LoS}}=\frac{1}{1+a\exp\left(-b\left[\theta_{m,n}-a\right]\right)},$$

where *a* and *b* are environmental parameters related to the building area, building density, building height, and corresponding distribution. $\theta_{m,n}$ is the elevation angle between UAV $U_{m}$ and user $I_{n}$, which is dependent on the height *h* of the UAV and the distance *d* between the user and the UAV. The expression is:

(2) $$\theta_{m,n}=\frac{180}{\pi }\arcsin\left(\frac{h}{d_{m,n}}\right).$$

The path loss of both line-of-sight and non-line-of-sight links between the UAV and the user is a combination of additional losses $\zeta_{\mathrm{LoS}}$ and $\zeta_{\mathrm{NLoS}}$ under the free-space transmission model.

(3) $$l_{m,n}^{\mathrm{LoS}}={\left(\frac{4\pi f_{c}d_{m,n}}{c}\right)}^{\alpha }\cdot 10^{\zeta_{\mathrm{LoS}}/10},$$

(4) $$l_{m,n}^{\mathrm{NLoS}}={\left(\frac{4\pi f_{c}d_{m,n}}{c}\right)}^{\alpha }\cdot 10^{\zeta_{\mathrm{NLoS}}/10},$$

where $f_{c}$ is the carrier frequency, *c* is the speed of light, and $\zeta_{\mathrm{LoS}}$ and $\zeta_{\mathrm{NLoS}}$ (in dB) are converted to linear scale via the exponential term. The average path loss can be expressed through a method that comprehensively considers both line-of-sight and non-line-of-sight conditions.

(5) $$PL_{m,n}=p_{m,n}^{\mathrm{LoS}}l_{m,n}^{\mathrm{LoS}}+\left(1-p_{m,n}^{\mathrm{LoS}}\right)l_{m,n}^{\mathrm{NLoS}}.$$

Therefore, the average channel gain between UAV $U_{m}$ and user $I_{n}$ is:

(6) $$G_{m,n}=\frac{1}{PL_{m,n}}.$$

In communication between UAVs and users, signal-to-noise ratio (SNR) is considered an important metric, hence the use of average channel gain to model it. The SNR of UAV-receiving signals from user devices is:

(7) $$\gamma_{m,n}=\frac{P_{n}G_{m,n}}{\sigma^{2}},$$

where $\sigma^{2}$ is the noise power, $P_{n}$ is the user’s transmit power, and $G_{m,n}$ is the average channel gain; $\sigma^{2}$ (and the SNR threshold $\gamma_{th}$ used below) are expressed in linear units, converted from the dBm/dB values used in the simulations.

According to Shannon’s channel capacity formula, the theoretical communication rate between UAV $U_{m}$ and user $I_{n}$ can be calculated as:

(8) $$R_{m,n}=b_{m,n}\log_{2}\left(1+\frac{P_{n}G_{m,n}}{\sigma^{2}}\right),$$

where $b_{m,n}$ is the bandwidth allocated by the UAV $U_{m}$ to user $I_{n}$. The total throughput $l_{m,n,T_{t}}$ within time slot τ between the UAV and the user can be represented as:

(9) $$l_{m,n,T_{t}}=\int_{T_{t}}^{T_{t}+\tau }b_{m,n}\log_{2}\left(1+\frac{P_{n}G_{m,n}}{\sigma^{2}}\right)dt.$$

### 3.2. Optimization Function

Our goal is to find the optimal association between UAVs and users to minimize communication energy consumption. To reduce algorithm complexity, we first cluster the users to form clusters equal in number to the UAVs. Then, association is performed based on reward functions.

For UAV, the design of the optimization function at the UAV side aims to maximize the aggregate intracluster throughput in the UAV swarms’ network. The benefit of UAV $U_{m}$ assisting user cluster $C_{k}$ can be expressed as:

(10) $$r_{m/k}=\sum_{n=1}^{N}\eta \times l_{m,n,T_{t}}\times x_{m,n},$$

where $x_{m,n}$ is a decision variable, which is set to 1 when user $I_{n}$ belongs to the serviced cluster $C_{k}$ by UAV $U_{m}$, and $x_{m,n}=0$ otherwise. *N* is the total number of users. η is the attenuation coefficient, and each UAV has its maximum number of assisted users, $Num_{m}$, which is set to ⌈N/M⌉ in the simulations. Exceeding this number may result in interference between users, leading to a decrease in overall performance. The expression is:

(11) $$\eta =\left\{\begin{array}{cc} 1 & \left|N_{k}\right|\leq Num_{m},\\ \frac{1}{\log\left(\left|N_{k}\right|-Num_{m}+e\right)} & \left|N_{k}\right|>Num_{m}. \end{array}\right.$$

$\left|N_{k}\right|$ represents the number of users contained in cluster $C_{k}$. Each UAV calculates its benefit value with respect to each user cluster and generates a preference list accordingly, where a higher ranking indicates a stronger preference for UAV to be matched with that user cluster.

For users, the design of the optimization function at the user end focuses on minimizing the total transmit power $P_{n}$. The benefit of user cluster $C_{k}$ associated with UAV $U_{m}$ can be expressed as:

(12) $$r_{k/m}=-\sum_{n=1}^{N}P_{n}\times x_{m,n},$$

where $x_{m,n}$ is a decision variable, which is set to 1 when user $I_{n}$ belongs to cluster $C_{k}$; otherwise, $x_{m,n}=0$. We aim to ensure that the signal-to-noise ratio for each user is greater than the threshold value $\gamma_{th}$.

(13) $$\frac{P_{n}G_{m,n}}{\sigma^{2}}\geq \gamma_{th}.$$

Solving the above inequality yields:

(14) $$P_{n}\geq \frac{\gamma_{th}\sigma^{2}}{G_{m,n}}.$$

Each user cluster calculates its benefit from being assisted by each UAV and generates a preference list accordingly, where a higher ranking indicates a stronger preference for the user cluster to be matched with that UAV.

## 4. User Clustering Based on Density Function

In this section, we describe the density function-based clustering algorithm used to solve the optimization problem.

Our proposed clustering algorithm consists of three stages: spatial gridding and data mapping phase, cluster center election phase, and cluster member assignment phase. In the spatial gridding and data mapping phase, the target dataset is converted from continuous space to discrete grid space, and point-based operations are converted to grid-based operations, which not only simplifies data processing but also reduces the computational burden in subsequent stages. In the second stage, cluster centers are selected by identifying density peaks based on the calculation of local density and relative distance; this step ensures that only the most representative and isolated high-density areas are selected as cluster centers, thereby promoting the accuracy and reliability of the clustering results. In the final stage, cluster membership assignment is performed through a mechanism of cluster formation, center adjustment, and re-clustering to balance cluster sizes and further refine network partitioning. The sequential design improves clustering accuracy and builds a balanced and structured clustering framework, laying a solid foundation for subsequent UAV–user association.

### 4.1. Spatial Gridding and Data Mapping Phase

The primary step of the spatial gridding and data mapping phase is to determine the size and boundaries of the grid based on the spatial distribution characteristics of the UAV–user clusters.

This involves dividing one dimension of the spatial region occupied by the user clusters into *p* grid cells. Each grid cell represents a semi-open interval in each dimension, with the specific interval range expressed as $\mathrm{Grid}=\left[m_{1},n_{1}\right),\left[m_{2},n_{2}\right),\ldots ,\left[m_{p},n_{p}\right)$. Uniform and non-uniform grid partitioning are two ways to partition the grid. Uniform grid partitioning provides an intuitive and computationally efficient method for clustering, effectively identifying cluster centers and suitable for most datasets. On the other hand, non-uniform grid partitioning has advantages in handling datasets with different density distributions, but quantifying the sparsity of data increases algorithm complexity and reduces its generality. Therefore, this paper adopted uniform grid partitioning for clustering analysis, ensuring that the length of the grid was the same in each dimension. This approach simplifies the computational process and ensures the effectiveness and applicability of clustering analysis; thus, each grid’s length is:

(15) $$d_{len}=\frac{x_{\max}-x_{\min}}{p},$$

where $x_{\max}$ and $x_{\min}$ represent the maximum and minimum coordinates of data points in the current dimension, respectively. Next, an index is assigned to each grid, denoted as $\mathrm{Grid}=\left(g_{1},g_{2},\ldots ,g_{n},\ldots \right)$. In this paper, the clustered users were all at the same altitude; thus, the system was established as a two-dimensional space. The grid scale was set to p=20; the parameter-sensitivity analysis in Section 6 shows that the throughput varies by less than 5% for *p* between 10 and 30, so the clustering outcome is insensitive to that choice. The decay factor $e^{-d_{ij}^{2}}$ in Equation (19) ensures that the main density information is captured by the grid user count $N_{g_{i}}$; the exponential term adds refinement from neighboring grids. The number of clusters *K* is set to K=M, since *K* must equal the number of available UAVs.

After the grid division, data mapping is required to assign each user to its corresponding grid. The two-dimensional vector for grid $g_{i}$ is represented by $\left(g_{i1},g_{i2}\right)$. For point *q*, it belongs to grid $g_{i}$ if and only if the following equation holds:

(16) $$g_{ij}=\left\lfloor \frac{q\left(j\right)-\min_{j}}{d_{len}}\right\rfloor ,$$

where $\left\lfloor x\right\rfloor$ denotes the floor function, q(j) represents the coordinate of point *q* in dimension *j*. $\min_{j}$ is the minimum value among all points in the set *Q* in dimension *j*, where j=1,2. Then, we establish coordinate axes for the area under analysis and assign a unique identifier to each non-empty grid, while also calculating the number of user nodes contained in each grid. This partition not only simplifies the spatial structure of the data but also facilitates subsequent density calculations and cluster analysis.

The expression for calculating the number of users $N_{g_{i}}$ in grid $g_{i}$ is:

(17) $$N_{g_{i}}=\sum_{q\in Q}u\left(q,g_{i}\right),$$

where *q* is the dataset of users, $u\left(q,g_{i}\right)$ represents the indicator function with a value of 1 if $q\in g_{i}$, and 0 otherwise. The expression for $u\left(q,g_{i}\right)$ is as follows:

(18) $$u\left(q,g_{i}\right)=\left\{\begin{array}{cc} 1 & q\in g_{i}\\ 0 & q\notin g_{i} \end{array}\right..$$

The spatial gridding and data mapping phase achieve the transformation of data point processing to grid point processing, simplifying the data structure and significantly reducing the computational burden during the algorithm’s execution process. This effectively manages and processes the dataset of users in the UAV swarms.

### 4.2. Clustering Center Election Phase

After completing the grid partitioning, the clustering algorithm for UAV–user clusters in this paper enters the core phase of clustering center election. In this stage, it is crucial to compute local density and relative distance, as these two indicators are essential for identifying clustering centers.

The local density measures the concentration of data points within a grid. In the clustering algorithm proposed in this paper, the local density of each grid is estimated by considering both the number of data points within the grid itself and the number of data points in neighboring grids affected by distance. This aims to more accurately reflect the data density of each grid, effectively identifying high-density areas and providing a basis for subsequent clustering center selection and data point allocation. The local density $\rho_{i}$ of grid $g_{i}$ is given by:

(19) $$\rho_{i}=N_{g_{i}}+\sum_{j\in Q_{i}}N_{g_{j}}\times e^{-d_{ij}^{2}},$$

where $Q_{i}$ denotes the set of adjacent non-empty grids to grid $g_{i}$, $N_{g_{i}}$ represents the number of users within the grid $g_{i}$, $e^{-d_{ij}^{2}}$ is the decay factor, which is related to the distance $d_{ij}$ between grids $g_{i}$ and $g_{j}$:

(20) $$d_{ij}=\sqrt{{\left({\mathrm{col}}_{i}-{\mathrm{col}}_{j}\right)}^{2}+{\left({\mathrm{row}}_{i}-{\mathrm{row}}_{j}\right)}^{2}},$$

where ${\mathrm{col}}_{i}$ and ${\mathrm{col}}_{j}$ are the column coordinates of grids $g_{i}$ and $g_{j}$, and ${\mathrm{row}}_{i}$ and ${\mathrm{row}}_{j}$ are the row coordinates of grids $g_{i}$ and $g_{j}$. In the process of determining clustering centers, grids with relatively large relative distances are considered potential candidates for clustering centers because they not only have higher local densities themselves but are also relatively independent from other high-density regions, with no other denser clusters around them. Then, we calculate the local density of all grids based on Equation (19) and compare them with those of other grids. Finally, we record the relative distance $\delta_{i}$ of grid $g_{i}$ to the grid with the highest local density in its neighborhood.

(21) $$\delta_{i}=\left\{\begin{array}{cc} \min_{j:\rho_{i}<\rho_{j}}d_{ij} & \;\mathrm{if}\;\rho_{i}<\rho_{j}\\ \max_{i\neq j}d_{ij} & \rho_{i}\;\mathrm{is}\;\mathrm{maximum}\; \end{array}\right.$$

In Equation (21), the grid points with the maximum local density are considered density peaks, as no other grid has a density exceeding them. To identify this special case, the algorithm sets the relative distance of such points to a maximum value. For other potential density peaks, they need to possess two characteristics simultaneously: a high local density value and a large relative distance. For grid $g_{i}$, its decision value $\gamma_{i}$ is defined as:

(22) $$\gamma_{i}=\rho_{i}\times \delta_{i}.$$

Finally, the grid point with the highest decision value, equal to the number of UAVs, is selected as the clustering center.

### 4.3. Cluster Member Assignment Phase

We analyze the quality of the clusters and decide whether adjustments are needed to further optimize the clustering structure and prevent significant differences between clusters. The expressions for the maximum and minimum cluster size thresholds $N_{\max}$ and $N_{\min}$ are as follows:

(23) $$\left\{\begin{array}{c} N_{\max}=\frac{N}{K}\left(\log_{10}\left(K\right)+1\right),\\ N_{\min}=\max\left(0,\frac{N}{K}\left(1-\log_{10}\left(K\right)\right)\right), \end{array}\right.$$

where *N* is the total number of users, and *K* is the number of clusters. If there exists a cluster with size $N_{k}>N_{\max}$, the users farthest from the cluster center, amounting to $N_{k}-N_{\max}$, are removed. Additionally, this cluster is then marked as full and no longer accepts any new users. The removed users are subsequently reassigned to the nearest clusters that are not marked as “full”. If there exists a cluster with size $N_{k}<N_{\min}$, users from the nearest clusters are moved in, amounting to $N_{\min}-N_{k}$. These steps are repeated until all cluster sizes meet the required criteria.

## 5. Matching Game and Algorithm

To address the optimal association between UAVs and users, in this section, we propose a user association scheme based on matching game theory.

In the deployment of UAV swarms’ networks, matching theory can be employed to achieve optimal associations based on the preferences of both parties, ensuring that each UAV serves the most suitable user cluster. The objective of matching theory is to attain a stable bipartite matching state, wherein under the given preference lists, for any UAV–user cluster pair, no alternative matching can be found that would yield higher satisfaction for both parties after rematching than their current satisfaction levels [35].

### 5.1. Game Formulation

The user association problem can be formulated as the following matching game:

- Players: Consist of unmanned aerial vehicles belonging to set $\left\{U_{1},U_{2},\ldots ,U_{M}\right\}$ and user clusters belonging to set $\left\{C_{1},C_{2},\ldots ,C_{K}\right\}$, where M=K.
- Strategy: Both the UAVs and the user clusters construct preference lists over the opposite side; in the deferred-acceptance procedure of Algorithm 1, the user clusters act as the proposing side, and each UAV either keeps or rejects the clusters in its request list to establish the match.
- Preference Lists: During the matching process, each unmanned aerial vehicle and user cluster have their own preferences and requirements. Unmanned aerial vehicles can assess and rank user clusters based on factors such as their geographic locations, number of users, and communication needs, while user clusters can evaluate and rank unmanned aerial vehicles based on their service capabilities.

**Algorithm 1** Intelligent networking algorithm with density-based clustering.  1:Initialize the system environment: each participant discovers its neighbors and collects the required network parameters.  2:**Phase I: User Clustering**  3:System spatial grid division.  4:Preprocess user data points and map them to the divided grid cells.  5:Calculate $\gamma_{i}$ for all grid points according to Equation (22).  6:Plot decision graph and select grid clustering centers.  7:Cluster the grid points based on the minimum distance principle, ensuring that user data points are consistent with the clusters to which their grid objects belong.  8:**for** each cluster $C_{K}$ **do**  9:     **if** $N_{k}>N_{\max}$ **then**10:         Remove the farthest $N_{k}-N_{\max}$ users and mark the cluster as “full”.11:         Assign the removed users to the nearest clusters that are not in the “full” state.12:     **end if**13:     **if** $N_{k}<N_{\min}$ **then**14:         Move in $N_{\min}-N_{k}$ users from the nearest other clusters.15:     **end if**16:**end for**17:**Phase II: Matching Game**18:**for** each UAV $U_{m}$ **do**19:     calculate $r_{m/k}$ on Equation (10), and establish a UAV’s preference lists.20:**end for**21:**for** each user cluster $C_{k}$ **do**22:     calculate $r_{k/m}$ on Equation (12), and establish a user cluster’s preference lists.23:**end for**24:**while** user clusters are not fully matched **do**25:     Unmatched user clusters apply to their most preferred UAV.26:     Each UAV selects the most preferred user cluster from the request list and rejects other clusters. ** **27:     Rejected user clusters remove the top-ranked UAV from their preference list.28:**end while**

The above model requires a precise and flexible matching mechanism to ensure efficient and fair pairing between unmanned aerial vehicles and users.

### 5.2. Preferences Lists

We use ${≿}_{m}$ to represent the preference ranking of unmanned aerial vehicles $U_{m}\in \left\{U_{1},U_{2},\ldots ,U_{M}\right\}$, and ${≿}_{k}$ to represent the preference ranking of user clusters $C_{k}\in \left\{C_{1},C_{2},\ldots ,C_{K}\right\}$.

UAVs evaluate all available user clusters and establish preference rankings based on the aggregate cluster throughput to determine which user cluster to request for association. In other words, the higher the aggregate cluster throughput, the higher the bandwidth utilization rate for the UAV, leading to greater system benefits. For a UAV $U_{m}$, its preference ranking ${≿}_{m}$ on user clusters is represented by:

(24) $$C_{k}{≿}_{m}C_{k}^{\prime }\Leftrightarrow r_{m}\left(C_{k}\right)\geq r_{m}\left(C_{k}^{\prime }\right),$$

where $r_{m}\left(\cdot \right)$ denotes the reward for UAV $U_{m}$, as given by Equation (10). $C_{k}{≿}_{m}C_{k}^{\prime }$ indicates that UAV $U_{m}$ prefers user cluster $C_{k}$ to $C_{k}^{\prime }$, or $C_{k}=C_{k}^{\prime }$.

User clusters evaluate all the applying UAVs, and based on the transmission power, establish preference ranking, given the premise of meeting service quality requirements. For a user cluster $C_{k}$, its preference ranking over UAVs is denoted as ${≿}_{k}$:

(25) $$U_{m}{≿}_{k}U_{m}^{\prime }\Leftrightarrow r_{k}\left(U_{m}\right)\geq r_{k}\left(U_{m}^{\prime }\right),$$

where $r_{k}\left(\cdot \right)$ denotes the reward of user cluster $C_{k}$, as given by Equation (12). $U_{m}{≿}_{k}U_{m}^{\prime }$ represents the preference of user cluster $C_{k}$ for UAV $U_{m}$ over $U_{m}^{\prime }$ or $U_{m}=U_{m}^{\prime }$.

### 5.3. Algorithm for User Association

In our proposed algorithm, both UAVs and users are considered rational and selfish. The core steps include initializing the system, constructing preference lists, performing matching iterations, and checking match stability. Through continuous iteration, the algorithm gradually converges to a stable bipartite matching strategy. The user-to-cluster association is many-to-one and is resolved by the density-based clustering stage. The cluster-to-UAV association, in contrast, is a one-to-one matching between K=M clusters and *M* UAVs, and the deferred-acceptance procedure operates on this one-to-one level: the user clusters act as the proposing side. The proposed matching procedure implements the Gale–Shapley deferred acceptance algorithm [36]: it terminates after at most M×K proposals and returns the stable matching most preferred by the proposing user clusters. Ties, if any, are broken by increasing cluster or UAV index, so the preference lists are strict and complete. This ensures that no pair of UAV–user clusters wishes to rematch with any other object outside the current match. The algorithm is described in Algorithm 1.

With the simulation parameters used in this work, the grid scale p=20 gives G=400 grid cells and $d_{len}=50$ m; the number of clusters is K=M=6. The deferred-acceptance matching terminates after at most M×K=36 proposals.

The overall complexity of the proposed algorithm is $O(N\cdot K+G^{2})$. Specifically, grid mapping takes O(N); the relative-distance computation for density-peak identification takes $O(G^{2})$ over the non-empty grid cells; assigning users and grids to the *K* cluster centers takes O(N+G·K); constructing the two-sided preference lists requires evaluating the user–UAV utility values for all relevant pairs, summing to O(N·M)=O(N·K) since K=M; and the deferred-acceptance procedure takes O(M·K) proposals. With K=M=6, the matching-stage cost is dominated by preference construction, and the leading terms reduce to $O(N\cdot K+G^{2})$.

## 6. Simulation Results

This section validates the effectiveness of the proposed algorithm through simulation experiments and compares it with existing classical solutions, highlighting the significant performance improvements of the new approach.

We considered users randomly distributed within a 1000 m × 1000 m geographical area, with six UAVs stationed at fixed positions in the air at a height of 150 m. The simulation parameters were set as shown in Table 1.

Since this work targeted unevenly distributed user devices, a hotspot scenario is also evaluated later in this section: 80% of the users followed a Gaussian distribution with standard deviation 90 m around a randomly located hotspot center, while the remaining 20% were uniformly scattered over the area. Unless otherwise stated, all results were averaged over 200 independent Monte Carlo runs under fixed random seeds; *N* ranged from 50 to 400.

**Figure 2:**
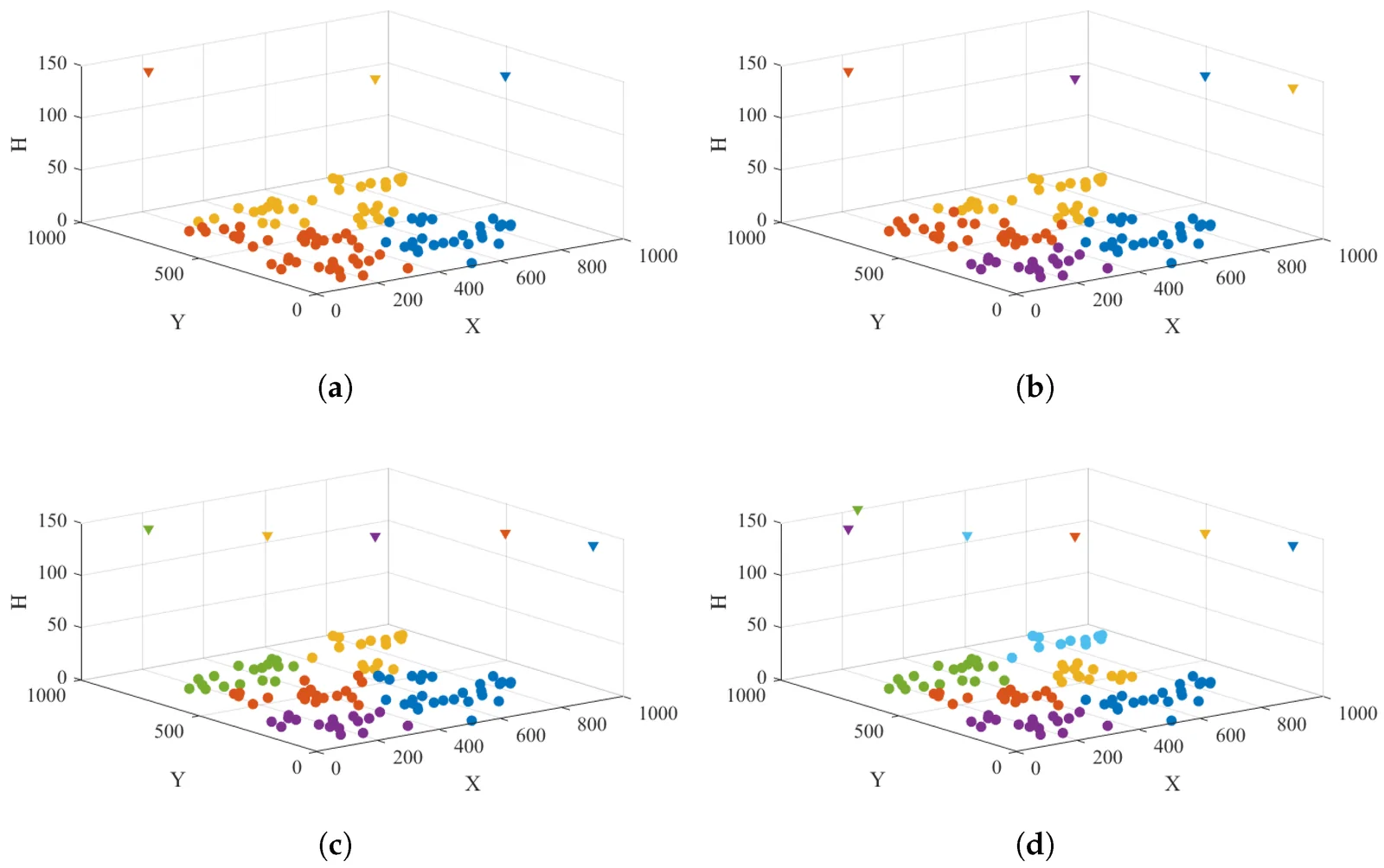
The association of UAVs and users. (**a**) n=3. (**b**) n=4. (**c**) n=5. (**d**) n=6. Circles denote user clusters and triangles denote UAVs; the same color indicates a matched UAV–user cluster pair.

Figure 2 shows the clustering results of the proposed algorithm with different numbers of UAVs. In this scenario, the UAV user devices are randomly deployed within a designated planar area, while several heterogeneous UAVs are positioned at fixed locations in the air. Figure 2 illustrates the matching results of UAVs and user clusters when the number of matches is n=3, n=4, n=5, and n=6, respectively. In these results, user clusters and UAVs with the same color indicate that they are matched with each other.

**Figure 3:**
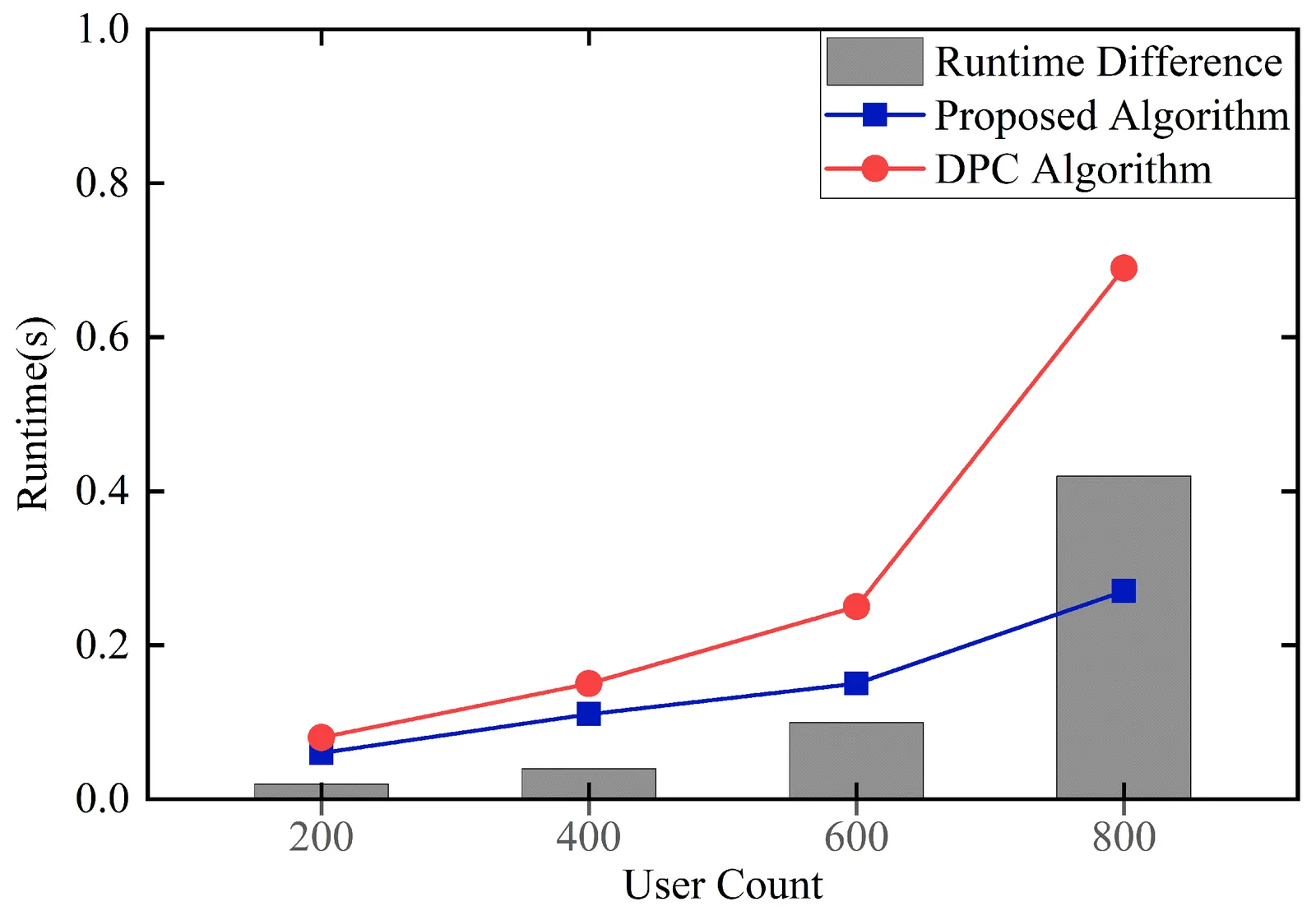
Comparison of runtime results.

Figure 3 compares the runtime of our algorithm with the density peak clustering (DPC) algorithm at user counts of 200, 400, 600, and 800 during the clustering phase. The DPC algorithm is a classical density peak clustering algorithm. When the number of users is small, the runtime of our algorithm is comparable to that of the DPC algorithm. However, as the number of users increases, the runtime difference between our algorithm and the DPC algorithm becomes larger. Particularly, at 800 users, our algorithm’s efficiency improves by 68% compared to the DPC algorithm. The simulation results validate the significant advantage of our proposed algorithm in improving computational efficiency. Theoretically, the proposed clustering achieves $O(N\cdot K+G^{2})$ complexity, where $G=p^{2}$ is the total number of grid cells, and provides an upper bound on the number of non-empty cells involved in the grid-level computation. In contrast, DPC requires $O(N^{2})$ pairwise distance computation, and K-means requires O(N·K·I) with *I* iterations. For space complexity, the proposed method stores grid counts and at most *K* cluster centers, i.e., O(N+G), while DPC maintains an N×N pairwise distance matrix, i.e., $O(N^{2})$.

Next, the communication performance of the algorithm was evaluated. To verify the effectiveness of the proposed algorithm, it was compared with other algorithms. The other algorithms included a random algorithm and a distance minimization algorithm, among other clustering-based baselines. The random algorithm randomly associated UAVs with users, while the distance minimization algorithm first calculated the distance matrix between users and UAVs and then associated them based on the shortest distance. In addition, we implemented K-means [37] and DPC [38], both paired with the same matching game, as clustering-based baselines. The comparison therefore focused on the three clustering-based schemes, with the random and distance-only algorithms serving as reference baselines. Here, the number of UAVs was set to six.

**Figure 4:**
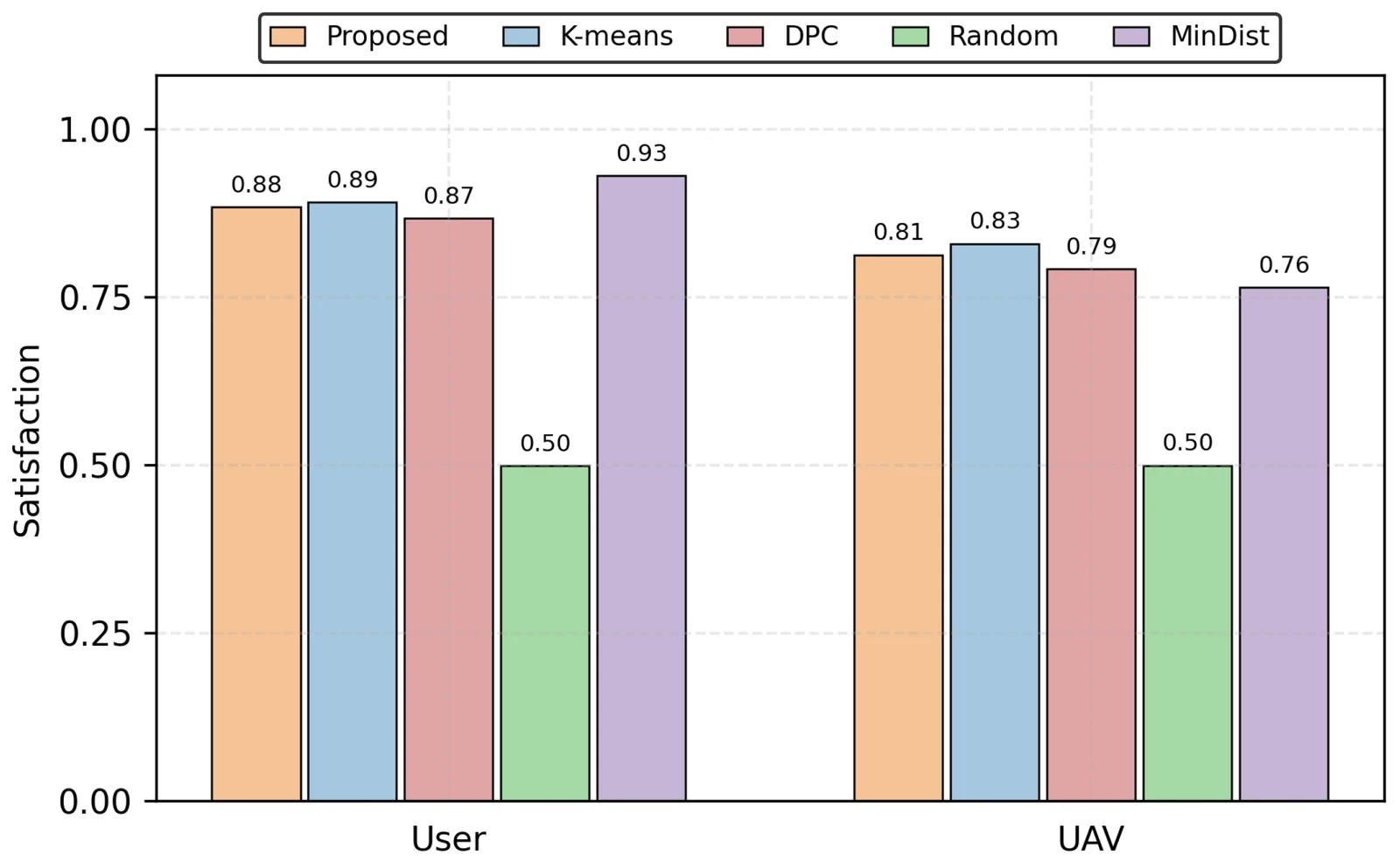
Satisfaction of different algorithms at N=200.

Figure 4 illustrates the satisfaction levels of users and UAVs obtained using different algorithms. Satisfaction was used to assess the quality of the UAV–user matching results, i.e., whether the preferences of UAVs and users were met. From the figure, it can be observed that satisfaction denotes the normalized preference rank of the matched partner, $S=1-\frac{r}{L-1}$, with rankings by channel gain and idle UAVs counted as zero. Proposed, K-means, and DPC achieve comparable satisfaction levels for both users and UAVs and all substantially outperform the random baseline: at N=200, the proposed algorithm reaches a user satisfaction of 0.88 and a UAV satisfaction of 0.81, improvements of 76% and 62% over the random algorithm, respectively.

**Figure 5:**
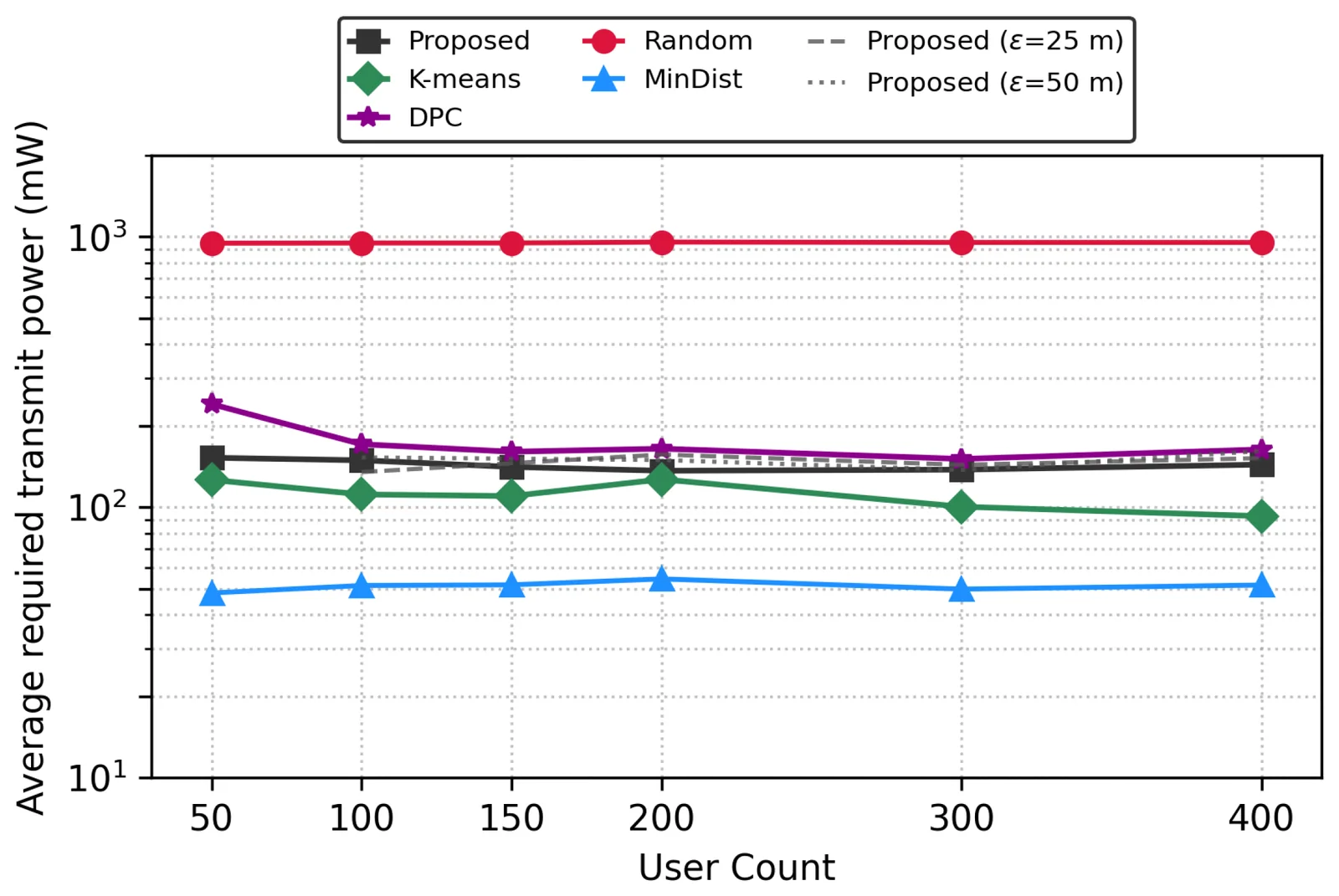
Average required transmit power per user in mW, logarithmic scale; the dashed (ϵ=25 m) and dotted (ϵ=50 m) curves show the proposed algorithm under user position estimation errors.

Figure 5 shows the comparison of different algorithms in terms of average required transmit power per user. The three clustering-based algorithms require substantially lower transmit power than the random baseline, with the proposed algorithm achieving an average required power of 143.3 mW per user across N=50–400, an 84.9% reduction compared to the random algorithm. The transmit power here denotes the minimum power required to satisfy the SNR threshold in Equation (14), plotted on a logarithmic axis. The lower power consumption comes from the density-based clustering, which groups users around the density peaks and shortens the average distance between UAVs and their assigned users.

The proposed algorithm is robust to user position estimation errors. The grid-based clustering quantizes user positions into cells of size $d_{len}$, so position errors affect the grid assignment primarily for users located near cell boundaries; grid quantization therefore reduces the sensitivity to position errors, though not entirely, and the clustering structure is mostly preserved. To quantify this robustness, we evaluated the algorithm under user position estimation errors of ϵ=5 to 50 m, where clustering and matching used the noisy positions; performance was then evaluated with the true positions. The throughput deviated by at most ±3.2% from the error-free case, and the user satisfaction decreased by no more than 1.3 percentage points; even at ϵ=50 m, equal to the grid size $d_{len}$, the throughput loss stayed below 3%. In Figure 5, the dashed (ϵ=25 m) and dotted (ϵ=50 m) curves depict the proposed algorithm under position errors, whose required power stays within approximately 15% of the error-free case for all evaluated user densities. The algorithm is also insensitive to the grid scale *p*: varying *p* from 10 to 30 changed the throughput by less than 5%, and no sharp performance drop occurred. The user satisfaction remained above 0.86 throughout, and the value p=20 adopted in this paper offered a good trade-off between density-estimation accuracy and computational cost. This quantitative sensitivity scan covered only the grid scale *p*; the number of clusters K=M was fixed by the number of UAVs and was not a free parameter, and the exponential decay term in the density estimate was set as described in Section 4.1 and was not scanned separately.

**Figure 6:**
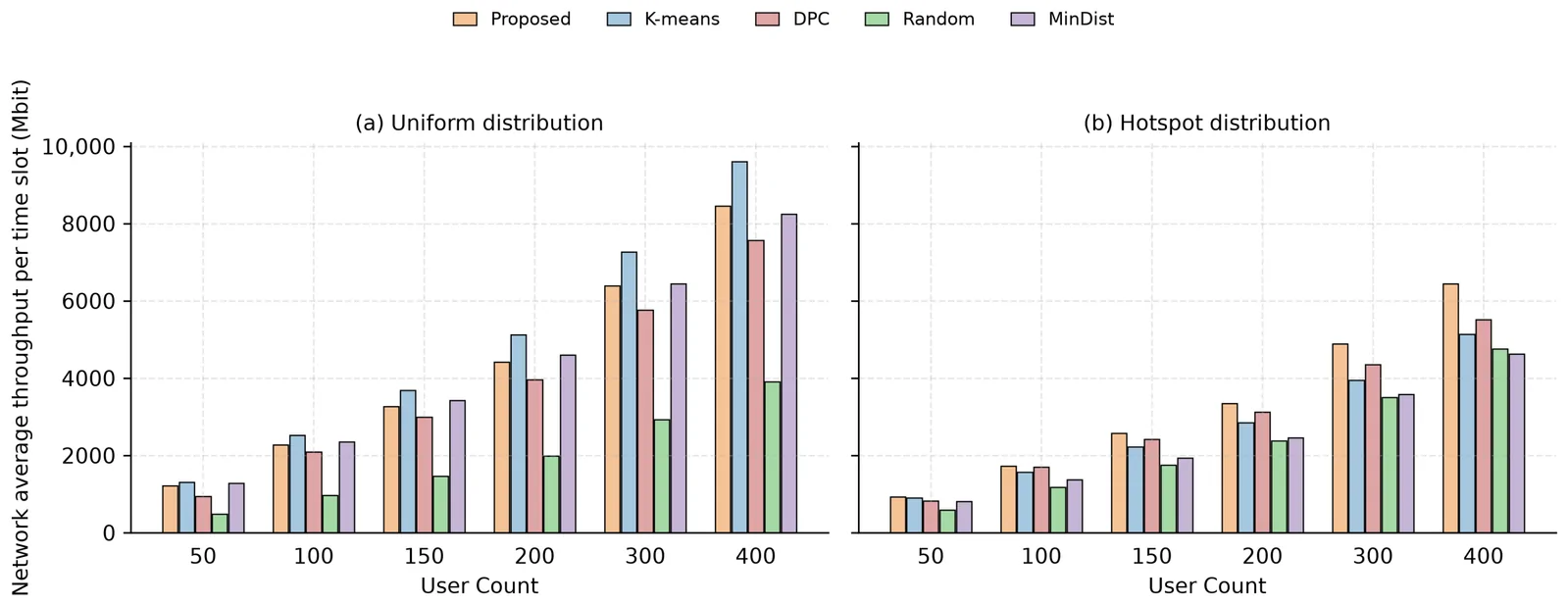
Network average throughput per time slot in Mbit, defined as the total delivered data of admitted users averaged over 200 Monte Carlo runs, under $P_{\max}=23$ dBm.

Figure 6 illustrates the network average throughput under different algorithms under a per-user power constraint $P_{\max}=23$ dBm, the 3rd Generation Partnership Project (3GPP) UE Power Class 3 limit [39]. It can be observed from the figure that the admitted-user throughput increases with the increase in the number of users. The admitted-user throughput denotes the total delivered data per time slot of admitted users, each admitted user contributing the fixed per-slot payload $B\log_{2}\left(1+\gamma_{th}\right)\cdot \tau$ with per-user bandwidth B=1 MHz, determined by the SNR threshold, averaged over the 200 Monte Carlo runs; users whose minimum required power exceeds $P_{\max}$ are not admitted. Under the uniform distribution, the three clustering-based algorithms all clearly exceed the random baseline, with K-means attaining the highest throughput and the proposed algorithm providing an average throughput of 4420.4 Mbit per time slot at N=200, a 122.5% improvement over the random algorithm. Since the three clustering-based schemes share the same matching game, the throughput differences among them stem from the clustering stage: density-based clustering forms more compact clusters, so the serving distances shorten and the per-user required transmit power drops (Figure 5). Under the power cap $P_{\max}$, this admits more users, and each admitted user contributes the fixed per-slot payload $B\log_{2}\left(1+\gamma_{th}\right)\cdot \tau$. Under the hotspot distribution (Figure 6b), the proposed algorithm also achieves the highest throughput among the five schemes, outperforming K-means and the random baseline at N=200 (3353.2 vs. 2856.2 and 2377.7 Mbit per time slot, i.e., by 17.4% and 41.0%, respectively); the advantage over K-means widens from 17.4% at N=200 to 25.4% at N=400, as denser users benefit more from compact clustering. The distance-only baseline performs on par with the clustering methods under the uniform distribution but falls clearly behind in the hotspot scenario, because it ignores user density.

## 7. Conclusions

This paper proposed an intelligent networking scheme for UAV-assisted network, reducing the average required transmit power per user and ensuring fast data transmission. Specifically, we analyzed and designed a density-based user clustering algorithm to address the issue of uneven user distribution, obtaining user clusters equal in number to the UAVs. Subsequently, we established a matching game, where UAVs and user clusters constructed preference lists based on their respective reward functions to achieve stable matching. Additionally, the throughput and transmission power of the UAV-assisted user system were analyzed. Simulation results demonstrated that the proposed intelligent matching and density-based clustering significantly improved system throughput and reduced the average required transmit power per user while achieving the highest throughput among the five compared schemes under hotspot user distributions and maintaining a low algorithm runtime. The algorithm is robust to user position errors and insensitive to the grid scale. The current framework assumes static users and UAVs within a single scheduling period, a simplification adopted to keep the algorithm design tractable. For mobile users, future work can update only the affected grids incrementally instead of recomputing each slot. Energy constraints can also be added to the matching objective. UAV mobility and time-varying channels remain outside the present static-association model; joint trajectory optimization and channel-aware preference updates are therefore important directions for future investigation.

## Figures and Tables

**Table 1:** Simulation parameters.

Parameter	Description	Value
*h*	UAV’s Altitude	150 m
*M*	Number of UAVs	6
*K*	Number of User Clusters	6
*p*	Grid Scale	20
$\sigma^{2}$	Noise Power	−109 dBm
*c*	Speed of Light	$3\times 10^{8}$ m/s
$f_{c}$	Carrier Frequency	2.4 GHz
α	Path Loss Exponent	2.3
$\gamma_{th}$	SNR Threshold	10 dB
τ	Time Slot	10 s
*a*	Environment Parameters	9.61
*b*	Environment Parameters	0.16
$\zeta_{\mathrm{LoS}}$	Additional Path Loss for LoS	3 dB
$\zeta_{\mathrm{NLoS}}$	Additional Path Loss for NLoS	20 dB

## Data Availability

The data presented in this study are available on request from the corresponding author.

## Abbreviations

The following abbreviations are used in this manuscript:

UAV	Unmanned Aerial Vehicle
IoT	Internet of Things
LoS	Line-of-Sight
NLoS	Non-Line-of-Sight
SNR	Signal-to-Noise Ratio
QoS	Quality of Service
DPC	Density Peak Clustering
SDN	Software-Defined Networking
